# Efficiency of Direct Transcutaneous Electroneurostimulation of the Median Nerve in the Regression of Residual Neurological Symptoms after Carpal Tunnel Decompression Surgery

**DOI:** 10.3390/biomedicines11092396

**Published:** 2023-08-27

**Authors:** Mustafa Al-Zamil, Inessa A. Minenko, Natalia G. Kulikova, Numman Mansur, Margarita B. Nuvakhova, Olga V. Khripunova, Irina P. Shurygina, Svetlana V. Topolyanskaya, Vera V. Trefilova, Marina M. Petrova, Ekaterina A. Narodova, Irina A. Soloveva, Regina F. Nasyrova, Natalia A. Shnayder

**Affiliations:** 1 Department of Physiotherapy, Faculty of Continuing Medical Education, Peoples’ Friendship University of Russia, 117198 Moscow, Russia; kulikovang777@mail.ru (N.G.K.); d-64-158@mail.ru (N.M.); 2 Department of Restorative Medicine and Neurorehabilitation, Medical Dental Institute, 127253 Moscow, Russia; kuz-inna@mail.ru; 3 Department of Sports Medicine and Medical Rehabilitation, I.M. Sechenov First Moscow State Medical University, 119991 Moscow, Russia; olaw@bk.ru; 4 National Medical Research Center for Rehabilitation and Balneology, 121099 Moscow, Russia; 1969margo@rambler.ru; 5 City Clinical Hospital Named after V. V. Vinogradov, 117292 Moscow, Russia; 6 Department of Ophthalmology, Rostov State Medical University, 344022 Rostov, Russia; ir.shur@yandex.ru; 7 Department of Hospital Therapy No. 2, I.M. Sechenov First Moscow State Medical University, 119991 Moscow, Russia; sshekshina@yahoo.com; 8 Institute of Personalized Psychiatry and Neurology, V.M. Bekhterev National Medical Research Centre for Psychiatry and Neurology, 192019 Saint Petersburg, Russia; vera.v.trefilova@yandex.ru (V.V.T.); nreginaf77@gmail.com (R.F.N.); 9 Shared Core Facilities “Molecular and Cell Technologies”, Professor V. F. Voino-Yasenetsky Krasnoyarsk State Medical University, 660022 Krasnoyarsk, Russia; stk99@yandex.ru (M.M.P.); katya_n2001@mail.ru (E.A.N.); solovieva.irina@inbox.ru (I.A.S.)

**Keywords:** carpal tunnel syndrome, carpal tunnel decompression surgery, transcutaneous electro-neurostimulation, Jebsen–Taylor hand function test, electromyography, negative sensory symptoms, Tinel’s symptom, Phalen’s symptom

## Abstract

Carpal tunnel syndrome (CTS) is the most frequent entrapment neuropathy. CTS therapy includes wrist immobilization, kinesiotherapy, non-steroidal anti-inflammatory drugs, carpal tunnel steroid injection, acupuncture, and physical therapy. Carpal tunnel decompression surgery (CTDS) is recommended after failure of conservative therapy. In many cases, neurological disorders continue despite CTDS. The aim of this study was to investigate the efficiency of direct transcutaneous electroneurostimulation (TENS) of the median nerve in the regression of residual neurological symptoms after CTDS. Material and Methods: 60 patients aged 28–62 years with persisting sensory and motor disorders after CTDS were studied; 15 patients received sham stimulation with a duration 30 min.; 15 patients received high-frequency low-amplitude TENS (HF TENS) with a duration 30 min; 15 patients received low-frequency high-amplitude TENS (LF TENS) with a duration 30 min; and 15 patients received a co-administration of HF TENS (with a duration of15 min) and LF TENS (with a duration of 15 min). Results: Our research showed that TENS significantly decreased the pain syndrome, sensory disorders, and motor deficits in the patients after CTDS. Predominantly, negative and positive sensory symptoms and the pain syndrome improved after the HF TENS course. Motor deficits, reduction of fine motor skill performance, electromyography changes, and affective responses to chronic pain syndrome regressed significantly after the LF TENS course. Co-administration of HF TENS and LF TENS was significantly more effective than use of sham stimulation, HF TENS, or LF TENS in patients with residual neurological symptoms after CTDS.

## 1. Introduction

Carpal tunnel decompression surgery (CTDS) is a treatment for carpal tunnel syndrome (CTS) via the surgical release of the transverse carpal ligament, thereby decompressing the median nerve [1]. The method was first described by Galloway in 1924 [2]. CTDS is recommended if conservative treatment fails and CTS symptoms persist or worsen [3]. The greatest effect of CTDS develops 6–12 months after the surgery [4,5]. Nocturnal numbness and tingling, with occasional daytime pain, persist after CTDS in 8.56% of patients [6,7]. The cause of residual neurological disorders after CTDS is incomplete surgical relaxation, due to the complexity of the surgical exploration of the distal transverse carpal ligament and the proximal antebrachial fascia [8]. In some cases, CTDS is not sufficiently effective, due to a long history of CTS before surgery, the presence of fibrous proliferation and tenosynovitis [9], or irreversible changes in the structures of the long-term compressed median nerve [10,11]. These conditions explain the persistence and severity of the residual neurological disorders of CTS in the early and late postoperative periods [12,13,14].

In many cases, revision surgery significantly improved the function of hands, with residual neurological symptoms after CTDS, by 33% at 6 months [15]. However, in 43–80% of patients, residual symptoms persisted 6 months postoperatively [8,16]. 

Conservative treatment of residual symptoms after CTDS begins with the use of central analgesics to relieve neuropathic pain syndrome [6]. The effectiveness of physiotherapy methods for the treatment of residual neurological symptoms after CTDS, using manual therapy [17,18], low-intensity laser therapy [19,20,21], acupuncture [22,23,24], and electroacupuncture [25], as well as extracorporeal shock wave therapy [26], has been proven in many studies. The effectiveness of these methods was of an individual nature—in some cases, they had a pronounced therapeutic effect, while in other cases, they were not sufficiently effective. In this regard, there remains a need to study and develop new effective physiotherapeutic methods for the treatment of this disease.

Transcutaneous electroneurostimulation (TENS) is a well-known method of physiotherapy [27,28]. Recent studies have helped clarify the mechanisms of the analgesic effect of TENS, which include stimulation of the release of endogenous endorphins [29], activation of opioid-mediated antinociception of rostroventromedial medulla and periaqueductal gray [30], activation of the descending inhibitory pathway [30,31,32], reduction of inflammation-induced sensitization of dorsal horn neurons [33], and acceleration of regenerative processes in damaged peripheral nerves [34] and denervation muscles [35,36].

In clinical practice, indirect TENS methods are more often used, in which sensory fibers and muscle fibers are stimulated in the areas of pain. However, in 2015, EMG-guided direct TENS of the peroneal and tibial nerves was proposed for the treatment of distal lower extremity polyneuropathy in patients with type 2 diabetes mellitus [37,38]. The efficiency of direct TENS exceeded the efficiency of indirect TENS by several times. At the same time, the improvement of the neurological symptoms of neuropathy was not limited to the analgesic effect, but extended to the regression of sensory and motor deficits and to the improvement of the EMG parameters of the affected nerves [37]. In subsequent studies, the direct TENS technique was applied in the treatment of patients with CTS in the preoperative period and demonstrated a significant regression of the pain syndrome, a decrease in sensory and motor deficits, and improvement in the EMG parameters of the affected median nerve [39].

The aim of this study was to investigate the efficiency of direct TENS of the median nerve in the regression of persisting neurological symptoms after CTDS. 

## 2. Materials and Methods

### 2.1. Participants

In this randomized controlled trial, we observed 60 patients (female—38, male—22) who had residual neurological symptoms of CTS for 6 to 9 months after CTDS.

The inclusion criteria for the study were as follows:

1. European patients;
2. Adult patients (male: 21 to 60 years old, female: 20 to 55 years old) according to World Health Organization classification;
3. Neurophysiological parameters of the median nerve without deterioration;
4. Amplitude compound muscle action potential of the median nerve > 2.5 mV;
5. History of CTS before CTDS of less than 5 years;
6. CTDS older than 6 months but less than 9 months;
7. The severity of paresthesia was 5 points or more;
8. Signed voluntary informed consent to participate in this study.

Exclusion criteria for the study were as follows:

1. Epilepsy and uncontrolled seizure disorder;
2. Severely cognitive disorders or mental illness;
3. Hereditary polyneuropathy or history of cervical radiculopathy C6;
4. Distal polyneuropathy of the upper extremities;
5. Damage to the median nerve anywhere along its path from the brachial plexus to the carpal canal;
6. History of stroke, spinal cord injury, traumatic brain injury, multiple sclerosis, or edema of the upper extremities;
7. History of cardiac arrythmias or hemodynamic instability;
8. Cardiac pacemaker or other implanted electronic system;
9. Botulinum toxin injections to any muscle of hand in the previous 3 months;
10. Evidence of deep venous thrombosis or other forms of venous thromboembolism;
11. Rheumatoid arthritis, gout, psoriasis, and arthrosis of the joints of the upper extremities;
12. Type 1 or type 2 diabetes mellitus;
13. Vascular atherosclerosis of the upper extremities;
14. Raynaud’s disease and vibration white finger;
15. De Quervain’s tenosynovitis, deformity and dislocation of the carpometacarpal joint of the thumb, and wrist arthritis;
16. Undergoing physiotherapy treatment or acupuncture after CTDS.

A “Voluntary Informed Consent in Research and Clinical Care” form was signed by all patients after explaining their state of health, the need for clinical, instrumental, and laboratory examination, and the proposed treatment, including possible complications or adverse events. The study protocol was approved by the local ethics medical committee (protocol No. 61, 3 September 2021). All procedures adhered to the 1984 Declaration of Helsinki and its later amendments. All patients read the above-named form in full (including the text, figures, and supplementary materials) and agreed to publication of the results.

Participation in this study was not rewarded. The researchers were not rewarded for their work. The study was carried out as part of a scientific research program of the Department of Physiotherapy of the Peoples’ Friendship University of Russia.

**Figure 1:**
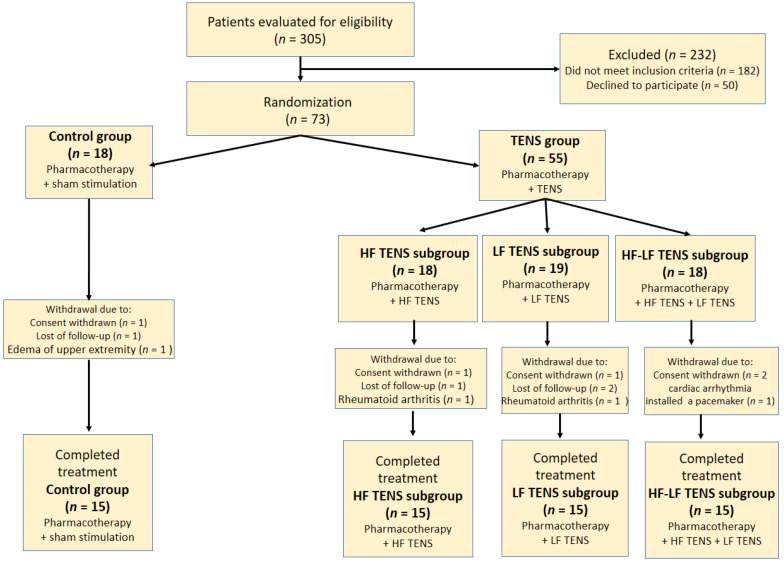
Flow chart of study population selection. Note: HF TENS—high-frequency low-amplitude transcutaneous electroneurostimulation; LF TENS—low-frequency high-amplitude transcutaneous electroneurostimulation; HF-LF TENS—combination of high-frequency low-amplitude transcutaneous electroneurostimulation and low-frequency high-amplitude transcutaneous electroneurostimulation.

The control group consisted of 15 patients (female—10, male—5). These patients received sham stimulation in addition to pharmacotherapy. The comparable (TENS) group consisted of 45 patients (female—28, male—17), including three subgroups, depending on the characteristics of direct TENS: subgroup 1 (HF TENS subgroup)—15 patients (female—9, male—6), co-administration of pharmacotherapy and HF TENS; subgroup 2 (LF TENS subgroup)—15 patients (female—10, male—5), co-administration of pharmacotherapy and LF TENS; subgroup 3 (HF-LF TENS subgroup)—15 patients (female—9, male—6) co-administration of pharmacotherapy and combined course of HF TENS and LF TENS. The female/male ratios were comparable in the control group, in the TENS group, and in all TENS subgroups (Figure 1). Thus, we compared four different methods of electrostimulation (sham stimulation and three TENS techniques) in the patients, who received the same pharmacotherapy prescribed by a neurologist.

The ages of the patients in the control group ranged from 36 to 65 years (mean age—49.8 ± 2.55 years). The ages of the patients in the HF TENS subgroup ranged from 35 to 63 years (mean age 49.1 ± 2.3 years). The ages of the patients in the LF TENS subgroup ranged from 34 to 65 years (mean age—50.2 ± 2.55 years). The ages of the patients in the HF- LF TENS subgroup ranged from 35 to 61 years (mean age—50.8 ± 1.82 years). The group–group differences in patients’ ages were not statistically significant (*p*-value > 0.1).

In the general sample, the patients suffered from CTS symptoms before CTDS from 2 to 5 years (mean duration—2.9 ± 0.09 years), as follows: control group—2.9 ± 0.23 years; HF TENS subgroup—2.9 ± 0.18 years; LF TENS subgroup—3.1 ± 0.18 years; HF-LF TENS subgroup—2.8 ± 0.14 years.

### 2.2. Sample Size Calculation

To determine the minimum number of subjects, we used the sample size calculator at ClinicCalc.com and Sealed Envelope online software (version 23). When searching the literature, it was found that in a study by Forst et al. [40], the total neuropathy symptom scores (NTSS-6) decreased after treatment of patients with diabetic neuropathy with active TENS by 42% and did not change in the control group after sham stimulation. The primary end point (measurement) that allows the minimum expected difference to be calculated is the anticipated percentage difference in the reduction of neuropathy symptoms according to the NTSS-6 questionnaire in the treatment group compared to the control group, which equals 42%. The power value = 80%. The expected significance level (*p*-value) = 0.05. When entering data, the sample s calculator of ClinicCalc.com and Sealed Envelope show that the minimum number of patients in each group is 15 patients. 

### 2.3. Neurological Examination

#### 2.3.1. Pain Syndrome

The pain syndrome was assessed using the visual analogue scale (VAS) and the Russian version of the McGill Pain Questionnaire (MPQ). The MPQ is composed of 78 words (descriptors), from which patients choose those words that best describe their experience of pain. The descriptors are summarized in 20 subclasses (subscales), which are distributed over three classes (dimensions): sensory (1st–14th subclasses); affective (15th–19th subclasses); and evaluative (20th subclass). In each subclass, descriptors are arranged in ascending order of synonymous value. Patients must select descriptors in any (not necessarily all) of the 20 subclasses, but only one descriptor per subclass. By summing values associated with each descriptor, the pain rating index (PRI) is subtracted. In this work, total PRI and the PRI for each dimension (sensory, affective, and evaluative) were used to assess the pain syndrome. 

#### 2.3.2. Positive Sensory Symptoms 

Patients determined the severity of tingling, numbness, and burning sensation in the wrist area and on the palmar surface of the affected hand on a 10-point scale. 

#### 2.3.3. Negative Sensory Symptoms 

**Figure 2:**
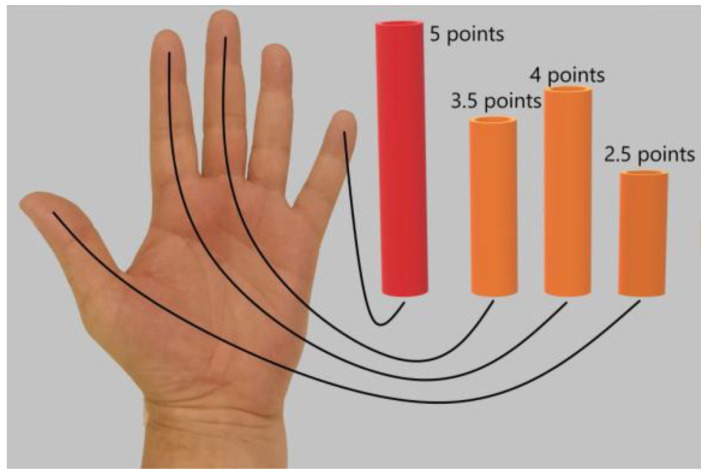
Determination of the severity of tactile sensation on the palmar surface of the thumb and the index and middle fingers relative to tactile sensation on the palmar surface of the little finger. The maximum level of tactile sensation on the palmar surface of the little finger was 5 points. In this case, the patient noted that the tactile sensation was 3.5/5 points on the middle finger, 4/5 points on the index finger, and 2.5/5 points on the thumb. For statistical processing, the average value of tactile sensation on three fingers was calculated as (3.5 + 4.0 + 2.5)/3 = 3.3 points.

The tactile sensation on the palmar surface of the thumb and the index and middle fingers of the affected hand was investigated using Semmes–Weinstein monofilament. The severity of tactile sensation was determined on a 5-point scale relative to the tactile sensation on the palmar surface of the little finger. The average of the values of tactile sensation determined on three fingers were calculated (Figure 2). 

#### 2.3.4. Motor Disorders

**Figure 3:**
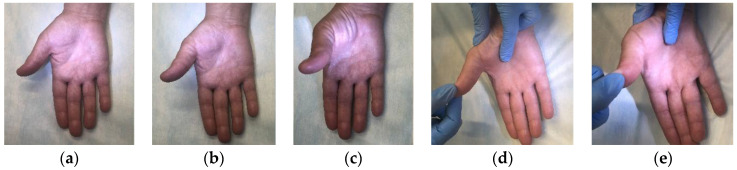
Muscle strength grading scale in m. abductor pollicis brevis. Note: (**a**) grade 1: muscle activation without full range of motion; (**b**) grade 2: muscle activation against gravity, full range of motion achieved; (**c**) grade 3: muscle activation against gravity, full range of motion achieved, (**d**) grade 4: muscle activation with some resistance, full range of motion achieved; (**e**) grade 5: muscle activation with full resistance by the examiner, full range of motion achieved.

To determine the dynamics of the motor function of the affected hand, the strength of m. abductor pollicis brevis was manually tested before and after treatment. The test technique included assessment of the muscle function in grades, depending on the strength of thumb abduction up and away from the plane of the palm. Muscle strength was assessed according to the muscle strength grading scale (Figure 3). Fine movement skills were studied using the Jebsen–Taylor test [41].

### 2.4. Neurophysiology Examination

The neurophysiological parameters of the median nerve were examined using evoked EMG studies of the median nerve. The sensory conduction velocity and the motor conduction velocity of the median nerve were measured bilaterally. The study was conducted before CTDS, 6 months after CTDS and 3 months after treatment. The motor-conduction velocity of the median nerve at the level of the brachial plexus, axillary region, shoulder, elbow, and forearm was examined. The latency of the compound muscle action potential (CMAP) was determined in the wrist area. The amplitude of the CMAP was measured in response to each stimulation of the nerve at each point. The sensory conduction velocity of the median nerve was studied antidromically through the carpal canal, and the amplitude of the sensory nerve action potential (SNAP) was determined. EMG examination was carried out on the EMG hardware complex (“Neuroexpeditor”, MBN company, registration certificate No. FSR 2010/07889). 

### 2.5. Ultrasonography of Carpal Canal 

In our study, all patients underwent an ultrasound diagnostic before treatment to exclude postoperative changes of the median nerve and surrounding tissues [42,43,44], but not all patients underwent an ultrasound diagnostic after treatment. Serious postoperative changes that required repeated surgical treatment were not detected in all patients. The studies were carried out on a Samsung Medison UGEO H60 ultrasound machine, registration number P3H 2013/691, dated 10 July 2017.

### 2.6. Pharmacotherapy

All patients underwent pharmacotherapy with the following drugs: alpha-lipoic acid—600 mg/day orally for 2 months; pentoxifylline—300 mg/day orally for 1 month; cyanocobalamin—1000 mcg/day (intramuscular injection) for 10 days; gabapentin—900 mg/day orally for 2 months. Pharmacotherapy began 1 month before TENS. In our study, no allergic reactions to the applied pharmacotherapy, nor any adverse effects, were observed.

### 2.7. Surgery of Carpal Tunnel Syndrome

All patients underwent traditional open carpal tunnel release with visualization of the carpal tunnel [45]. In all patients, surgical interventions were performed successfully without the development of complications or adverse events. CTS was diagnosed by a neurologist and confirmed by median-nerve EMG and carpal-tunnel ultrasound in all patients in the preoperative period. 

### 2.8. Transcutaneous Electroneurostimulation 

#### 2.8.1. Protocol of Sham Stimulation 

A cathode of 1 cm^2^ was fixed at the level of the wrist above the projection of the entrance of the median nerve into the carpal tunnel. A 1 cm^2^ anode was fixed on the distal part of the index finger. The current characteristics were as follows: monophasic rectangular with a frequency of 1 Hz; duration—50 μs; stimulation amplitude—1 mA above the initial sensory response. Stimulation was administered for 30 min. The number of procedures was 15. The procedures were carried out every other day.

#### 2.8.2. Protocol of High-Frequency Low-Amplitude Transcutaneous Electroneurostimulation 

A 1 cm^2^ anode was fixed at the level of the wrist above the projection of the entrance of the median nerve into the carpal canal. The 1 cm^2^ cathode (pen electrode) was left unfixed. The thumb and the index finger, middle finger, and ring finger were stimulated sequentially. The current characteristics were as follows: monophasic, rectangular with a frequency of 100 Hz; duration—100 μs; and the stimulation amplitude was raised until a comfortable painless sensory response was achieved. Amplitude adjustment was carried out for each finger separately. Each finger was stimulated for 10 s. The total amount of stimulation with each procedure was 30 min. The number of procedures was 15. The procedures were carried out every other day.

#### 2.8.3. Protocol of Low-Frequency High-Amplitude Transcutaneous Electroneurostimulation

**Figure 4:**
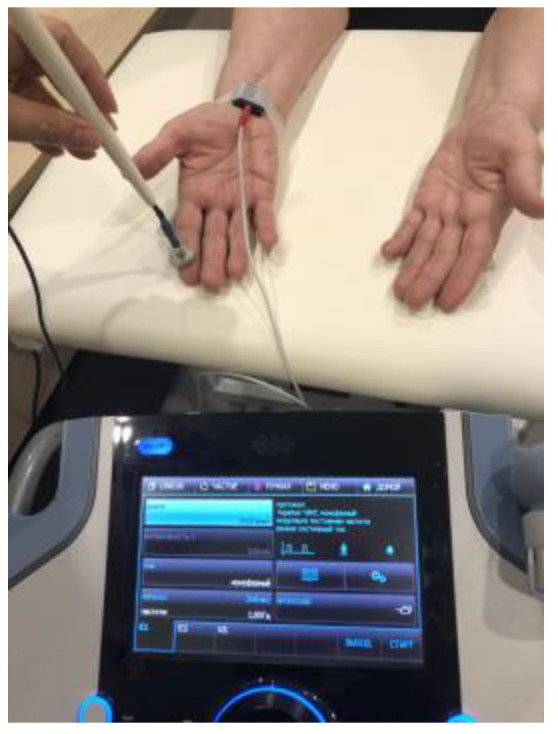
Stimulation of the median nerve by low-frequency high-amplitude transcutaneous electroneurostimulation. The cathode was fixed above the carpal channel. The anode (pen electrode) was not fixed. The thumb and the index, middle, and ring fingers were stimulated sequentially every 10 s.

A cathode of 1 cm^2^ was fixed at the level of the wrist above the projection of the entrance of the median nerve into the carpal tunnel. The 1 cm^2^ anode (pen electrode) was left unfixed. The thumb and the index finger, middle finger, and ring finger were stimulated sequentially (Figure 4). The current characteristics were as follows: monophasic, rectangular with a frequency of 1 Hz; duration—200 μs; and the stimulation amplitude was raised until a comfortable painless motor response was achieved. Amplitude adjustment was carried out for each finger separately. Each finger was stimulated for 10 s. The total amount of stimulation with each procedure was 30 min. The number of procedures was 15. The procedures were carried out every other day.

#### 2.8.4. Protocol of Co-Administration of High-Frequency Low-Amplitude Transcutaneous Electroneurostimulation and Low-Frequency High-Amplitude Transcutaneous Electroneurostimulation

For the electrical stimulation of the median nerve, a combined application of HF-TENS (stimulation duration of 15 min) and LF-TENS (stimulation duration of 15 min) was used. The total duration of the procedure was 30 min. The number of procedures was 15. The procedures were carried out every other day.

#### 2.8.5. Transcutaneous Electroneurostimulation Technique

A CE0434 certified BL-4000 smart/premium device (BL Industries Ltd., Hertfordshire, United Kingdom) was used for TENS (registration number RAN 2020/12648, dated 24 November 2020). The equipment has been used in Russia since 2010 (registration number FEZ 2010/06686, dated 29 April 2010).

### 2.9. Statistical Analysis

Collected data were computed and analyzed using SPSS Statistics for Windows, Version 25.0 (IBM SPSS Statistics for Windows, Version 25.0. Armonk, NY, USA: IBM Corp). Descriptive statistics were used to measure the means (M), standard deviation (SD), and standard error mean (SEM). Normality was tested using the Shapiro–WilK normality test. Levene’s test was used to assess the equality of variances between two or more groups. Multivariate analysis of variance (ANOVA) was used to test for differences between the means of the control group and the three TENS subgroups. To prevent data from incorrectly appearing to be statistically significant, the Bonferroni test was used. To compare the means of the same variable between two groups, we used an independent group *t*-test. The *p*-value < 0.05 provided evidence against the null hypothesis, suggesting a statistically significant effect.

## 3. Results

### 3.1. Assessment of Pain Syndrome

#### 3.1.1. Visual Analogue Scale 

**Figure 5:**
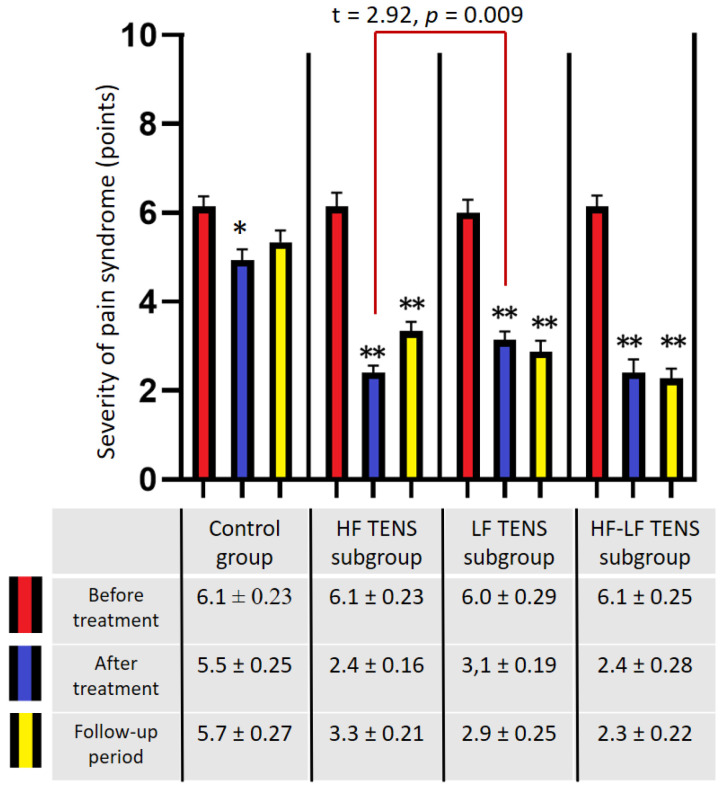
Dynamics of pain by visual analogue scale in patients with residual neurological symptoms after carpal tunnel decompression surgery). Note: * *p*-value < 0.05, ** *p*-value < 0.01; HF TENS—high-frequency low-amplitude transcutaneous electroneurostimulation; LF TENS—low-frequency high-amplitude transcutaneous electroneurostimulation; HF-LF TENS—combination of high-frequency low-amplitude transcutaneous electroneurostimulation and low-frequency high-amplitude transcutaneous electroneurostimulation.

Before treatment, in all groups, the pain syndrome exceeded 5 points and averaged 6.1 ± 0.1 points (Figure 5). The mean values in the control group and the TENS subgroups did not differ significantly (*p*-value > 1). After treatment, the analgesic effect was 29.5% (t = 5.00, *p*-value = 0.0001) in the control group, 60.7% (t = 12.47, *p*-value = 0.0001) in the HF TENS subgroup, 48.3% (t = 8.36, *p*-value = 0.0001) in the LF TENS subgroup, and 60.7% (t = 9.86, *p* = 0.0001) in the HF-LF TENS subgroup. Overall, post-treatment pain in the TENS group was 38.8% (t = 5.02, *p*-value = 0.0001) less than it was in the control group. In addition, it was shown that the post-treatment pain syndrome was significantly lower it was in the HF TENS and HF-LF TENS subgroups than it was in the LF TENS subgroup, by 22.5% (t = 2.92, *p*-value = 0.009).

After a 3 month follow-up period, the level of pain in the control group did not change significantly (*p*-value > 1.0). However, the patients in the HF TENS subgroup showed an increase in pain by 37.5%, compared with the level of pain immediately after treatment (t = 3.41, *p*-value = 0.002). Despite this, the achieved analgesic effect was 45% less than the initial level of pain before treatment (t = 8.58, *p*-value = 0.0001). No significant changes were found in the levels of pain in the LF TENS and the HF-LF TENS subgroups compared with the achieved analgesic effect immediately after treatment (*p*-value > 0.1). 

#### 3.1.2. Pain Assessment Using McGill Pain Questionnaire

Before treatment, the results of a pain assessment using MPQ demonstrated no significant differences in total PRI in the control group and the TENS subgroups (*p*-value > 0.1). The average severity of the pain syndrome was 11.4 ± 0.18 points in the sensory dimension, 7.2 ± 0.11 points in the affective dimension, and 3.6 ± 0.09 points in the evaluative dimension.

*Immediately after treatment:*

**Figure 6:**
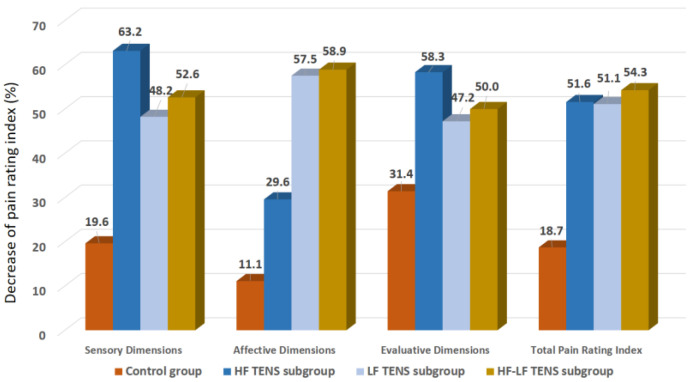
Decrease in pain rating index of the McGill Pain Questionnaire after treatment compared to baseline, in percentage. Note: HF TENS—high-frequency low-amplitude transcutaneous electroneurostimulation; LF TENS—low-frequency high-amplitude transcutaneous electroneurostimulation; HF-LF TENS—co-administration of high-frequency low-amplitude transcutaneous and low-frequency high-amplitude transcutaneous electroneurostimulation.

In the control group, significant decreases in the level of the pain syndrome were found in the sensory, affective, and evaluative dimensions, of 19.6% (t = 3.7, *p*-value = 0.01), 11.1% (t = 2.5, *p*-value = 0.03), and 31.4% (t = 16.7, *p*-value = 0.0001), respectively (Figure 6). In the TENS group, a significant decrease in the pain syndrome was registered, which turned out to be lower than the initial level by 52.3% (t = 16.7, *p*-value = 0.0001), including the sensory dimension—51.8% (t = 3.5, *p*-value = 0.001), the affective dimension—53.4% (t = 3.9, *p*-value = 0.001), and the evaluative dimension—51.9% (t = 4.2, *p*-value = 0.0003). However, the analgesic effect in the control group was less than it was in the TENS subgroups, by 38.9% (t = 5.7, *p*-value = 0.0001) in the sensory dimension, by 47.4% (t = 8.47, *p*-value = 0.0001) in the affective dimension, by 27.8% (t = 4.7, *p*-value = 0.001) in the evaluative dimension, and by 40.5% (t = 6.7, *p*-value = 0.0001) in the total PRI of the MPQ score. 

A comparative analysis among the TENS subgroups demonstrated that the reduction in PRI of the sensory dimension in patients treated with HF TENS was 12% better than that of patients treated with LF TENS (t = 2.47, *p* = 0.02). However, no significant differences between HF TENS and HF-LF TENS subgroups were found in the pain level after treatment (*p*-value > 0.1).

The PRI of the affective dimension decreased, mainly, in the LF TENS and the HF-LF TENS subgroups, compared to the HF TENS subgroup, by 22.5% (t = 3.43, *p*-value = 0.003) and 25% (t = 3.80, *p*-value = 0.0007), respectively.

The regression of the PRI of the evaluative dimension turned out to be 21% more significant in the HF TENS subgroup than in the LF TENS subgroup (t = 2.44, *p*-value = 0.021), but no significant difference was found between the HF-TENS and the HF-LF TENS subgroups (*p*-value > 0.1).

The total PRI in all the TENS subgroups did not differ significantly after treatment (*p*-value > 0.1).

*After a 3 month follow-up period:*

The PRI of the sensory dimension did not change significantly, compared to the achieved therapeutic effect immediately after treatment in the control group (*p*-value > 0.1). However, in the HF TENS subgroup, an increase in the pain syndrome of 25.0% was determined (t = 3.29, *p*-value = 0.003), but the level of the pain syndrome did not change significantly in patients after LF TENS and HF-LF TENS (*p*-value > 0.1). A comparative analysis showed that the PRI of the sensory dimension was lower in HF-LF TENS subgroup than in the HF TENS subgroup, by 15.4% (t = 2.30. *p*-value = 0.03). 

The PRI of the affective dimension increased in the HF TENS subgroup by 37.5% (t = 6.30, *p*-value = 0.0001), but the PRI of the affective dimension continued to decrease in the LF TENS and HF-LF TENS subgroups, by 9.7% and 13.3%, respectively. In the LF TENS and HF-LF TENS subgroups, the PRI of the affective dimension was significantly lower than it was in the HF TENS subgroup, by 49% (t = 13.1, *p*-value = 0.0001) and 53% (t = 13.6, *p*-value = 0.0001), respectively.

Negative dynamics of the PRI evaluative dimension were noted in the HF TENS subgroup, amounting to 40% (t = 6.60, *p*-value = 0.0001). However, a comparative analysis showed that the decreases in this indicator were 41.7% (t = 6.64, *p*-value = 0.0001) compared to baseline (before treatment) and 19.2% (t = 3.60, *p*-value = 0.014) compared to the control group after treatment. The maximum decrease in the PRI evaluative dimension was found in the HF-LF TENS subgroup, amounting to 23.8% (t = 2.60, *p*-value = 0.001), compared to HF TENS subgroup.

In the control group, the PRI regression of the evaluative dimensions was 25.7% (r = 4.38, *p*-value = 0.0001). 

### 3.2. Assessment of Positive Sensory 

**Figure 7:**
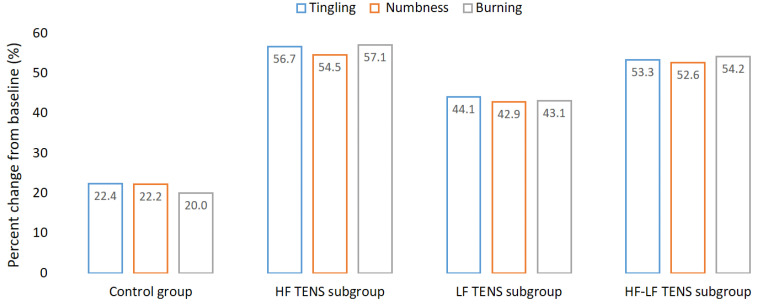
Dynamics of tingling, numbness, and burning after treatment, compared to the baseline before treatment. Note: HF TENS—high-frequency low-amplitude transcutaneous electroneurostimulation; LF TENS—low-frequency high-amplitude transcutaneous electroneurostimulation; HF-LF TENS—co-administration of high-frequency low-amplitude transcutaneous and low-frequency high-amplitude transcutaneous electroneurostimulation.

The mean level of tingling, numbness, and burning of the positive sensory symptoms in the affected hand after CTDS was 5.48 ± 0.12 points, according to VAS. There was no significant difference between control group and TENS subgroups in the severity of the positive sensory symptoms. After treatment, the mean regression of the positive sensory symptoms in the control group was 21.6% (t = 3.46, *p*-value = 0.002). The mean regression of the positive sensory symptoms in the TENS group was 50.9% (t= 8.07, *p*-value = 0.0001). At the same time, the severity of the positive sensory symptoms in the TENS group was less than it was in the control group, by 36.2% (t = 4.32, *p*-value = 0.0002). When comparing the TENS subgroups with each other, it was found that the best reduction in the positive sensory symptoms was in the HF TENS and HF-LF TENS subgroups, by 56.1% (t = 8.83, *p*-value = 0.0001) and 53.3% (t = 8.36, *p*-value = 0.0001), respectively. The regression of the positive sensory symptoms in the LF TENS subgroup was less than it was in the HF TENS and HF-LF TENS subgroups, by 23.4% (t = 2.74, *p*-value = 0.01) and 18.1% (t = 2.11, *p*-value = 0.04), respectively (Figure 7). 

At the end of the follow-up period, the regression of the positive sensory symptoms in the TENS subgroups persisted for 3 months. 

### 3.3. Assessment of Tactile Sensation 

Before treatment, no significant differences between the control group and the TENS subgroups in tactile sensation were found (*p*-value > 1.0). The mean level of tactile sensation was 3.5 ± 0.16 points in the control group, 3.4 ± 0.15 points in the HF TENS subgroup, 3.4 ± 0.15 points in the LF TENS subgroup, and 3.4 ± 0.16 points in the HF-LF TENS subgroup. 

After treatment, in the control group, sham stimulation did not lead to a significant improvement in the level of tactile sensation (*p*-value > 1.0). A significant improvement in tactile sensation was noted in all TENS subgroups: in the HF TENS subgroup—26.5% (t = 4.5; *p*-value = 0.0003); in the LF TENS subgroup—14.7% (t = 2.50, *p*-value = 0.02); and in the HF-LF TENS subgroup—20.6% (t = 3.39, *p*-value = 0.002). Improvement in tactile sensation was higher in the HF TENS subgroup than it was in the LF TENS subgroup, by 10.3% (t = 2.26, *p*-value = 0.03). There was no statistically significant difference between the HF TENS and HF-LF TENS subgroups in tactile sensation (*p*-value > 0.1).

**Figure 8:**
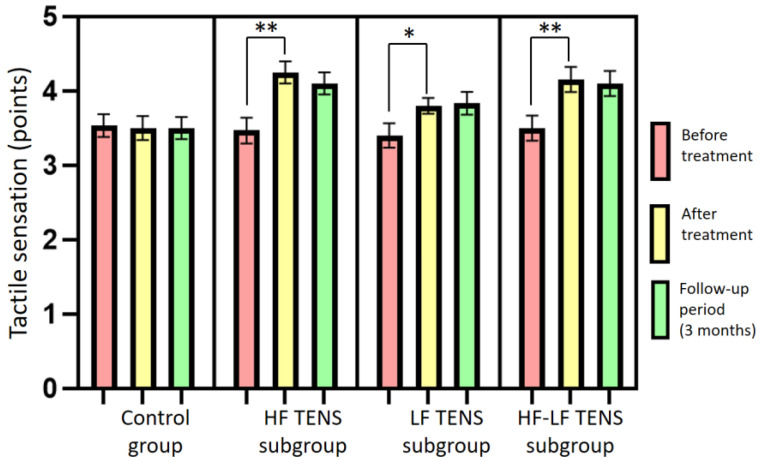
Dynamics of the tactile sensation levels in the affected hand after treatment in the control group and the TENS subgroups (mean ± SEM). Note: * *p*-value < 0.05, ** *p*-value < 0.01; HF TENS: high-frequency low-amplitude transcutaneous electroneurostimulation; LF TENS: low-frequency high-amplitude transcutaneous electroneurostimulation; HF-LF TENS: co-administration of high-frequency low-amplitude transcutaneous electroneurostimulation and low-frequency high-amplitude transcutaneous electroneurostimulation.

After 3 months of follow-up, the improvement in tactile sensation levels that was achieved after TENS treatment remained unchanged in all TENS subgroups (*p*-value > 1.0) (Figure 8). 

### 3.4. Assessment of Muscle Strength in the Affected Hand 

Before treatment, the mean strength levels of m. abductor pollicis brevis were 3.75 ± 0.11 grade in the control group and 3.75 ± 0.11 grade in the TENS group, without significant group–group differences (*p*-value > 1.0).

**Figure 9:**
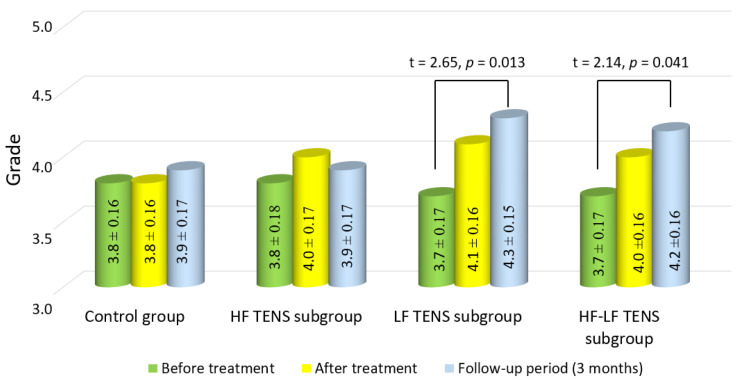
Dynamics of muscle strength of m. abductor pollicis brevis in the affected hand after treatment in the control group and the TENS subgroups (mean ± SEM). Note: HF TENS—high-frequency low-amplitude transcutaneous electroneurostimulation; LF TENS—low-frequency high-amplitude transcutaneous electroneurostimulation; HF-LF TENS—co-administration of high-frequency low-amplitude transcutaneous electroneurostimulation and low- frequency high-amplitude transcutaneous electroneurostimulation.

After treatment, in the control and TENS groups, significant changes in the strength of m. abductor pollicis brevis were not observed. However, there was a moderate but non-significant increase in muscle strength in the LF TENS and HF-LF TENS subgroups, of 10.8% and 8.1%, respectively (*p*-value > 0.1). After 3 months of follow-up, the improvement in the muscle-strength levels of m. abductor pollicis brevis in the LF TENS and HF-LF TENS subgroups were significant, compared to the control group, amounting to 16.2% and 13.5%, respectively (*p*-value < 0.05). No significant differences between these subgroups wre found (*p*-value > 1.0) (Figure 9).

### 3.5. Assessment of Fine Movement Skills by the Jebsen–Taylor Test 

Before treatment, the mean duration of the Jebsen–Taylor test required to complete the task was extended in all patients (Table 1), as follows: writing a short sentence—16.8 ± 0.14 s.; turning card—9.6 ± 0.11 s.; picking up small common objects—8.8 ± 0.10 s.; simulated feeding—8.6 ± 0.10 s.; stacking checkers up to 5.6 ± 0.10 s.; picking up and transferring large light objects—5.9 ± 0.09 s.; picking up and transferring large heavy objects—7.6 ± 0.10 s. The mean duration of this test in all sub-tests was 66% longer (*p*-value < 0.01) than the normal duration [46]. There were no significant group–group differences in the duration of the Jebsen–Taylor test before the start of the study (*p*-value > 1.0).

**Figure 10:**
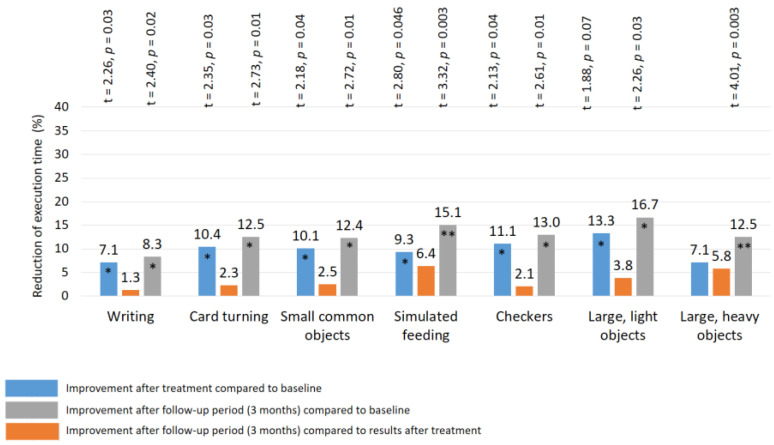
Improvement of fine movement skills according to the Jebsen–Taylor test in the HF TENS subgroup. Note: HF TENS—high-frequency low-amplitude transcutaneous electroneurostimulation; *—*p*-value < 0.05; **—*p*-value < 0.01.

**Figure 11:**
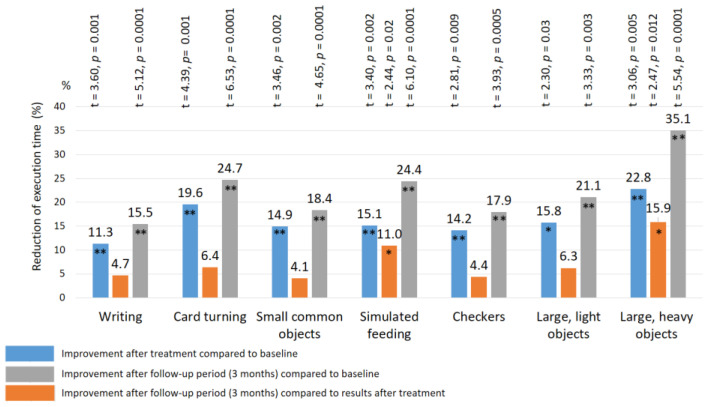
Improvement of fine movement skills according to the Jebsen–Taylor rest in the LF TENS subgroup. Note: LF TENS—low frequency high amplitude transcutaneous electroneurostimulation; *—*p*-value < 0.05; **—*p*-value < 0.01.

**Figure 12:**
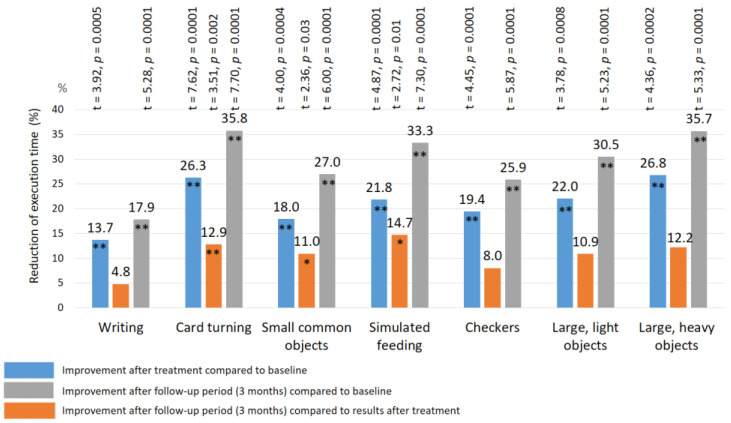
Improvement of fine movement skills according to the Jebsen–Taylor test in the subgroup with co-administration of HF TENS and LF TENS. Note: HF TENS—high-frequency low-amplitude subcutaneous electroneurostimulation; LF TENS—low-frequency high-amplitude transcutaneous electroneurostimulation; * *p*-value < 0.05; ** *p*-value < 0.01.

After treatment: a significant improvement in the Jebsen–Taylor test scores was found in all TENS subgroups, but not in the control group (Figure 10 and Figure 11). The total test-execution time in the HF TENS subgroup decreased by 9.8% (t = 2.09, *p*-value = 0.046). However, a more pronounced decrease in test-execution time was demonstrated in the LF TENS and HF-LF TENS subgroups—15% (t = 3.16, *p*-value = 0.004) and 19.3% (t = 4.10, *p*-value = 0.0003), respectively (Figure 12). The patients in the HF-LF TENS subgroups performed this test faster than the patients of the HF-TENS subgroup, by 10.8% (t = 2.5, *p*-value = 0.01). However, no significant differences between the LF TENS and the HF-LF TENS subgroups in test-execution time were found (*p*-value > 0.1). In the control group, the Jebsen–Taylor test did not change significantly in any of the sub-tests (*p*-value > 1.0).

After the 3 month follow-up period, no significant changes were found in the HF-TENS subgroup. However, if we compare these results with the baseline data before treatment, we find an additional positive therapeutic response in the reduction of the test duration in all sub-tests, by 12.6% (t = 4.91, *p*-value = 0.001). 

In the LF TENS subgroup, the positive dynamics continued with improvement in the simulated-feeding sub-test by 11% (t = 2.44, *p* = 0.02) and in the picking-up-large-light-objects sub-test by 15.9% (t = 2.47, *p* = 0.012). The total time to complete all sub-tests, compared with the initial results (before treatment), significantly decreased, by 20.4% (t = 4.65, *p*-value = 0.0001).

In the HF-LF TENS subgroup, an 11% (t = 2.31, *p*-value = 0.03) decrease in the mean duration of the Jebsen–Taylor test was registered, compared to post-treatment outcomes, and there was a 28.1% (t = 5.438, *p*-value = 0.0001) decrease, compared to the initial duration (before treatment).

### 3.6. Electrodiagnostic Evaluation of Median Nerve

The mean latency and amplitude of the CMAP, the sensory conduction velocity and the SNAP did not differ significantly between all studied groups (*p*-value > 0.01). After treatment, the latency and the amplitude of the CMAP, the sensory conduction velocity, and the SNAP did not change significantly in any of the studied groups (*p*-value > 0.1). At the same time, the latency of the CMAP in five patients of the LF TENS subgroup and in four patients of the HF-LF TENS subgroup decreased by 1.3 ± 0.18 s and 1.2 ± 0.22 s, respectively. However, the CMAP amplitude increased by 1.15 ± 0.22 mV in four patients in the LF TENS subgroup and by 1.10 ± 0.20 mV in four patients in the HF-LF TENS subgroup. 

## 4. Discussion

Our study demonstrated that the treatment of residual neurological symptoms after CTDS, by a combination of TENS and pharmacotherapy, was more pronounced than it was with the use of pharmacotherapy alone.

The analgesic effect of TENS immediately after treatment exceeded the analgesic effect of pharmacotherapy by 91.6% (VAS) and by 65% (MPQ), compared with the preservation of the analgesic effect during the first 3 months of the follow-up period. The severity of spontaneous positive sensory symptoms (paresthesia) reduced by 1.35 times after TENS treatment and by 1.12 times at the end of the follow-up period. Compared to pharmacotherapy, TENS decreased the levels of positive sensory symptoms by 50.9% after treatment and by 49.9% at the end of the follow-up period. A prolonged decrease of 20.6% in the severity of tactile hypothesis occurred b in the TENS group, but there was no significant change after the use of pharmacotherapy alone. An increase of 14.9% in the motor function of the abductor pollicis brevis was significant after TENS but did not change after the use of pharmacotherapy alone. Improvement in movement skills and fine motor skills of the hand, as determined by the Jebsen–Taylor test, was found only after TENS—it was 15.5% after treatment and 21.6% at the end of the follow-up period. Electromyography of the affected median nerve showed a significant increase in the CMAP amplitude by 1.13 mV and a decrease in the CMAP latency by 1.25 s in 20% of cases after TENS, but there were no changes after pharmacotherapy.

A comparative analysis between the HF TENS and LF TENS subgroups demonstrated that the severity of the pain syndrome after treatment was 22.6% less in the HF TENS subgroup than it was in the LF TENS subgroup. Using MPQ, we demonstrated that the sensory dimensions of the pain syndrome decreased more after HF TENS than after LF TENS, by 30.9%. In addition, the affective dimensions of the pain syndrome turned out to be 38% less after LF TENS and by 49% less at the end of follow-up period, compared with HF TENS. The effectiveness of HF TENS in reducing the positive sensory symptoms (tingling, numbness, and burning) was 29.4% higher than that of LF TENS. However, at the end of the follow-up period, the average level of the positive sensory symptoms after HF TENS and LF TENS did not differ significantly. HF TENS was 79.6% more effective in improving the tactile sensation of the median nerve than LF TENS immediately after treatment and at the end of the follow-up period. The recovery of the motor function of m. abductor pollicis brevis was detected in patients after LF TENS but not after HF TENS. In addition, the improvement in fine motor skills of the hand was higher in patients after LF TENS than it was in patients after HF TENS immediately after treatment, by 49%, and at the end of the follow-up period, by 56%. Improvement in the EMG parameters of the affected median nerve was registered in 30% of patients after LF TENS, but this was not observed in patients after HF TENS.

The results of our study demonstrated the high efficacy of combining two modalities of median nerve electrostimulation (LF TENS and HF TENS) in the treatment of patients with residual neurological symptoms after CTDS. A feature of the application of this method, in comparison with the use of the individual modalities of HF TENS and LF TENS, is the expansion of the range of therapeutic effect. At the same time, the maximum sensory recovery, analgesic, and anti-participate effects of HF TENS and the maximum motor recovery, anti-inflammatory, and anti-effective effects of LF TENS were achieved. Similar results were found in previous studies, where the maximal effectiveness of TENS on the symptoms of distal polyneuropathy in patients with type 2 diabetes mellitus was registered after the combined use of HF TENS and LF TENS [37,38]. Furthermore, the high efficiency of combined HF TENS and LF TENS was proven in many experimental [47,48] and clinical studies [32,37,38]. However, in this work, for the first time, we proved the high efficiency of combined HF and LF TENS in the treatment of residual neurological symptoms after CT, compared with the use of separate HF TENS and LF TENS

The analgesic effect of TENS can be achieved at several analgesic levels, including the segmental, supplemental, and local levels [32]. The segmental analgesic mechanism is due to the neurotic stimulation effect of electrical impulses on the A-e sensory fibers of the median nerve. Excitation of these fibers activates the pain gate mechanism in the dorsal horns of the spinal cord to inhibit the transition of pain signals from the noxious A-e and C fibers to the brain, and thus reduces the sensation of pain [28,30,31]. The segmental analgesic effect is more characteristic of HF TENS, since the stimulation of fast fibers with TENS at a frequency of 100 Hz is 100 times stronger than their stimulation with TENS at a frequency of 1 Hz. This explains the rapid and marked reduction in pain immediately after HF TENS, compared the reduction after LF TENS. The frequency of electrical impulses during HF TENS is tens of times higher than the frequency of LF TENS pulses, but the difference in the analgesic effect of these techniques did not exceed 31%. This discrepancy is explained by other analgesic mechanisms of LF TENS, which simultaneously act along with the segmental mechanism as parasegmental and local analgesic mechanisms. 

A parasegmental analgesic mechanism is carried out mainly by activating of collateralized periaqueductal gray [29,30] and the suprasegmental pathways of β-endorphin and met-enkephalin [49,50]. At the same time, the analgesic effect correlates most of all with the painless current amplitude and the pulse duration [51]. This mechanism is more typical for LF TENS. Another insufficiently studied suprasegmental analgesic mechanism of LF TENS is the reflex acupuncture effect. LF TENS is also known as acupuncture, as is TENS [52]. Some authors believe that the mechanism of action of acupuncture is associated with the direct stimulation of ergo-receptors and the excitation of muscle afferent (Aδ) fibers, which cause stimulation of the brain in its various parts, especially in the hippocampus [53]. A characteristic of this mechanism is the arrival of impulses to the brain parasegmentally (without the participation of the spinal cord). As a result, the secretion of many different anti-stress neurotransmitters is stimulated, such as serotonin, norepinephrine, substance P, dopamine, β-endorphin, enkephalin, dynorphin, and neuropeptide Y. The release of these neurotransmitters in response to LF TENS reduces the severity of the affective response of the brain to persistent neuropathic pain [54,55,56,57,58]. Local analgesic mechanisms in the zone of TENS application are associated with improved microcirculation [59,60], accumulation of anti-inflammatory and analgesic substances, and reduction in proinflammatory cytokines [61,62]. Another important mechanism is the local effect of HF TENS and LF TENS on degenerative changes that develop in the median nerve and the surrounding tissues [63,64,65]. As a result of the stimulation of regenerative processes in the affected area, the restoration of damaged tissues is accelerated, the afferentation of nociceptive receptors decreases, and spontaneous ectopic activity reduces, which decreases the severity of peripheral sensitization.

In all of the methods of electrostimulation of the median nerve (HF TENS, LF TENS, and HF-LF TENS) we used, the analgesic effect persisted for 3 months after the end of the course of treatment, which can be explained by the temporary deactivation of the foci of peripheral and central sensitization [37,38,39,66,67,68,69]. The prolonged effect of TENS was observed not only in the treatment of pain, but also extended to the reduction of sensory and motor deficits. The persistent positive remission of sensory and motor disorders within 3 months after the end of treatment indicates restoration in the structure of the nerve, as well as the development of persistent compensatory re-innervation changes in the affected muscles. In the literature, the prolonged effect of TENS has been demonstrated in many works [70,71,72].

## 5. Limitations

1. We did not have the opportunity to study the morphological changes of the median nerves using ultrasonography after TENS treatment, because in our study we used only clinical and neurophysiological methods of examination to appraise the effectiveness of treatment. In the next studies, we plan to conduct a control examination of TENS treatment of residual neurological symptoms after CTDS with ultrasonography.
2. We were unable to compare the efficacy of TENS with other physical therapy treatments. First, this was not the purpose of our study, and second, we compared the effectiveness of TENS with a (sham stimulation) placebo. The effectiveness of various physiotherapeutic methods, such as manual therapy [17,18], low-intensity laser therapy [19,20,21], acupuncture [22,23,24], electroacupuncture [25], and extracorporeal shock wave therapy [26] has been proven in many studies in the treatment of patients with residual neurological symptoms after CTDS, but TENS has never been studied in the treatment of this disease. Thus, before comparing TENS with other methods of treatment, it was necessary first to prove the effectiveness of TENS in the treatment of patients with residual neurological symptoms after CTDS with the determination of optimal current parameters and appropriate modalities to achieve the maximum therapeutic effect. However, in the next works, we plan to compare TENS with ultrasound and laser therapy in the treatment of patients with residual neurological symptoms after CTDS.

## 6. Conclusions

Our research showed that TENS, but not sham stimulation, significantly decreased the pain syndrome, sensory disorders, and motor deficits in patients with residual neurological symptoms after CTDS. Negative and positive sensory symptoms and pain decreased after co-administration of pharmacotherapy and HF TENS. The best regression of motor deficits, reduction of fine motor skill performance, electromyography changes, and affective responses to chronic pain syndrome in these patients occurred after the co-administration of pharmacotherapy and LF TENS. The co-administration of HF TENS and LF TENS was significantly more effective than the separate use of HF TENS or LF TENS in expanding the range of therapeutic effects, with a pronounced analgesic effect and significant recovery of sensory and motor functions in the patients with residual neurological symptoms after CTDS.

## 7. Declaration of Patient Consent

The authors confirm that they have obtained all necessary patient consent forms. In the forms, the patients provided their consent for the publication of their images and other clinical information in the journal. The patients understood that their names and initials would not be published and that appropriate steps would be taken to conceal their identities, but that anonymity could not be guaranteed.

## Figures and Tables

**Table 1:** Assessment of fine movement skills by the Jebsen–Taylor test (mean ± SEM, s.).

Reflex	Control Group			TENS Group									
				HF TENS Subgroup			LF TENS Subgroup			HF-LF TENS Subgroup			Normal [46]
Writing	16.9± 0.49	16.2 ± 0.46	17.2 ± 0.48	16.8 ± 0.45	15.6 ± 0.38 *	15.4 ± 0.37 *	16.8 ± 0.41	14.9 ± 0.33 **	14.2 ± 0.30 **	16.8 ± 0.47	14.5 ± 0.35 **	13.8 ± 0.32 **	11.7 ± 2.1
Card turning	9.4 ± 0.28	9.1 ± 0.26	9.5 ± 0.28	9.6 ± 0.30	8.6 ± 0.30 *	8.4 ± 0.32 *	9.7 ± 0.32	7.8 ± 0.29 **	7.3 ± 0.18 **	9.5 ± 0.26	7.0 ± 0.20 **	6.1 ± 0.16 **##	4.3 ± 1.4
Small common objects	8.7 ± 0.30	8.5 ± 0.28	9.2 ± 0.30	8.9 ± 0.26	8.0 ± 0.32 *	7.8 ± 0.31 *	8.7 ± 0.28	7.4 ± 0.25 **	7.1 ± 0.20 **	8.9 ± 0.32	7.3 ± 0.24 **	6.5 ± 0.24 **#	5.5 ± 1.0
Simulated feeding	8.4 ± 0.32	8.5 ± 0.30	8.2 ± 0.30	8.6 ± 0.30	7.8 ± 0.24 *	7.3 ± 0.25 *	8.6 ± 0.28	7.3 ± 0.26 **	6.5 ± 0.20 **#	8.7 ± 0.30	6.8 ± 0.25 **	5.8 ± 0.26 **#	6.7 ± 1.1
Checkers	5.5 ± 0.38	5.7 ± 0.35	5.6 ± 0.37	5.8 ± 0.35	4.9 ± 0.25 *	4.7 ± 0.26 *	5.4 ± 0.34	4.3 ± 0.30 **	4.1 ± 0.20 **	5.7 ± 0.30	4.5 ± 0.25 **	4.1 ± 0.20 **	3.3 ± 0.6
Large light objects	5.9 ± 0.30	6.0 ± 0.32	5.8 ± 0.30	6.0 ± 0.30	5.2 ± 0.30 *	5.0 ± 0.28 *	5.7 ± 0.31	4.8 ± 0.25 *	4.5 ± 0.20 **	5.9 ± 0.28	4.6 ± 0.20 **	4.1 ± 0.20 **	3.1 ± 0.5
Large heavy objects	5.4 ± 0.29	5.1 ± 0.28	5.2 ± 0.30	5.6 ± 0.30	5.2 ± 0.30	4.9 ± 0.25 **	5.7 ± 0.32	4.4 ± 0.28 **	3.7 ± 0.22 **#	5.6 ± 0.28	4.1 ± 0.20 **	3.6 ± 0.25 **	3.2 ± 0.5

Note: HF TENS—high-frequency low-amplitude transcutaneous electroneurostimulation; LF TENS—low-frequency high-amplitude transcutaneous electroneurostimulation; HF-LF TENS—co-administration of high-frequency low-amplitude transcutaneous electroneurostimulation and low-frequency high-amplitude transcutaneous electroneurostimulation. Significant reduction time compared with the initial results (before treatment): * *p*-value < 0.05; ** *p*-value < 0.01. Significant reduction time compared with the results after treatment: # *p*-value < 0.05; ## *p*-value < 0.01.

## Data Availability

Not applicable. Data is not available due to confidentiality and ethical restrictions.

## References

[1] Erfanifam T., Anaraki P.H., Vahedi L., Nourmohammadi J., Emami B., Khameneh A. (2022). The outcomes of carpal tunnel decompression based on electro-diagnostic approaches and clinical symptoms in patients suffering from carpal tunnel syndrome (CTS). J. Family Med. Prim. Care..

[2] Amadio P.C. (1995). The first carpal tunnel release?. J. Hand Surg. Br..

[3] Orhurhu V., Orman S., Peck J., Urits I., Orhurhu M.S., Jones M.R., Manchikanti L., Kaye A.D., Odonkor C., Hirji S. (2020). Carpal tunnel release surgery—A systematic review of open and endoscopic approaches. Anesth. Pain Med..

[4] Ise M., Saito T., Katayama Y., Nakahara R., Shimamura Y., Hamada M., Senda M., Ozaki T. (2021). Relationship between clinical outcomes and nerve conduction studies before and after surgery in patients with carpal tunnel syndrome. BMC Musculoskelet Disord..

[5] Guyette T.M., Wilgis E.F. (2004). Timing of improvement after carpal tunnel release. J Surg Orthop Adv..

[6] Aydin M., Argun G., Acar B., Arikan M., Toğral G., Cinaroglu S., Mert A., Demi Rtas M. (2021). Residual symptoms after carpal tunnel decompression and treatment with gabapentin: A multicenter study. Cureus.

[7] Botte M.J., von Schroeder H.P., Abrams R.A., Gellman H. (1996). Recurrent carpal tunnel syndrome. Hand Clin..

[8] Jones N.F., Ahn H.C., Eo S. (2012). Revision surgery for persistent and recurrent carpal tunnel syndrome and for failed carpal tunnel release. Plast. Reconstr. Surg..

[9] Campagna R., Pessis E., Feydy A., Guerini H., Le Vie D., Corlobé P., Drapé J.L. (2009). MRI assessment of recurrent carpal tunnel syndrome after open surgical release of the median nerve. AJR Am. J. Roentgenol..

[10] Al-Zamil M.K. (2022). Differential diagnosis of carpal tunnel syndrome. Bull. Med. Stomatol. Inst..

[11] Sevy J.O., Varacallo M. (2023). Carpal Tunnel Syndrome. 2022. StatPearls [Internet].

[12] Kronlage S.C., Menendez M.E. (2015). The benefit of carpal tunnel release in patients with electrophysiologically moderate and severe disease. J. Hand Surg. Am..

[13] Fowler J.R., Munsch M., Huang Y., Hagberg W.C., Imbriglia J.E. (2016). Pre-operative electrodiagnostic testing predicts time to resolution of symptoms after carpal tunnel release. J. Hand Surg..

[14] Matsis R., Chou J., Clode N. (2020). Outcome of carpal tunnel decompression with pre-surgical diagnosis determined on general practitioner assessment and nerve conduction study. J. Clin. Orthop. Trauma..

[15] Sun P.O., Selles R.W., Jansen M.C., Slijper H.P., Ulrich D.J.O., Walbeehm E.T. (2019). Recurrent and persistent carpal tunnel syndrome: Predicting clinical outcome of revision surgery. J. Neurosurg..

[16] Kilinc F., Behmanesh B., Seifert V., Marquardt G. (2021). Does Recurrence of Carpal Tunnel Syndrome (CTS) after Complete Division of the Transverse Ligament Really Exist?. J. Clin. Med..

[17] Mohamed F.I., Hassan A.A., Abdel-Magied R.A., Wageh R.N. (2016). Manual therapy intervention in the treatment of patients with carpal tunnel syndrome: Median nerve mobilization versus medical treatment. Egypt. Rheumatol. Rehabil..

[18] Wolny T., Linek P. (2019). Is manual therapy based on neurodynamic techniques effective in the treatment of carpal tunnel syndrome? A randomized controlled trial. Clin. Rehabil..

[19] Pratelli E., Pintucci M., Cultrera P., Baldini E., Stecco A., Petrocelli A., Pasquetti P. (2015). Conservative treatment of carpal tunnel syndrome: Comparison between laser therapy and Fascial Manipulation((R)). J. Bodyw. Mov. Ther..

[20] Chang W.D., Wu J.H., Jiang J.A., Yeh C.Y., Tsai C.T. (2008). Carpal tunnel syndrome treated with a diode laser: A controlled treatment of the transverse carpal ligament. Photomed. Laser Surg..

[21] Güner A., Altan L., Kasapoğlu Aksoy M. (2018). The effectiveness of the low-power laser and kinesiotaping in the treatment of carpal tunnel syndrome, a pilot study. Rheumatol. Int..

[22] Cabýoglu M.T., Ergene N., Tan U. (2006). The mechanism of acupuncture and clinical applications. Int. J. Neurosci..

[23] Bahrami-Taghanaki H., Azizi H., Hasanabadi H., Jokar M.H., Iranmanesh A., Khorsand-Vakilzadeh A., Badiee-Aval S. (2020). Acupuncture for Carpal Tunnel Syndrome: A Randomized Controlled Trial Studying Changes in Clinical Symptoms and Electrodiagnostic Tests. Altern. Ther. Health Med..

[24] Ho C.Y., Lin H.C., Lee Y.C., Chou L.W., Kuo T.W., Chang H.W., Chen Y.S., Lo S.F. (2014). Clinical effectiveness of acupuncture for carpal tunnel syndrome. Am. J. Chin. Med..

[25] Li T., Yan J., Hu J., Liu X., Wang F. (2022). Efficacy and safety of electroacupuncture for carpal tunnel syndrome (CTS): A systematic review and meta-analysis of randomized controlled trials. Front. Surg..

[26] Kim J.C., Jung S.H., Lee S.U., Lee S.Y. (2019). Effect of extracorporeal shockwave therapy on carpal tunnel syndrome: A systematic review and meta-analysis of randomized controlled trials. Medicine.

[27] Chapman R.C., Wilson M.E., Gehrig J.D. (1976). Comparative effects of acupuncture and transcutaneous stimulation on the perception of painful dental stimuli. Pain.

[28] Melzack R., Wall P.D. (1984). Acupuncture and transcutaneous electrical nerve stimulation. Postgrad. Med. J..

[29] Sluka K.A., Deacon M., Stibal A., Strissel S., Terpstra A. (1999). Spinal blockade of opioid receptors prevents the analgesia produced by TENS in arthritic rats. J. Pharmacol. Exp. Ther..

[30] DeSantana J.M., Da Silva L.F., De Resende M.A., Sluka K.A. (2009). Transcutaneous electrical nerve stimulation at both high and low frequencies activates ventrolateral periaqueductal grey to decrease mechanical hyperalgesia in arthritic rats. Neuroscience.

[31] Vance C.G., Dailey D.L., Rakel B.A., Sluka K.A. (2014). Using TENS for pain control: The state of the evidence. Pain Manag..

[32] Johnson M.I., Bjordal J.M. (2011). Transcutaneous electrical nerve stimulation for the management of painful conditions: Focus on neuropathic pain. Expert. Rev. Neurother..

[33] Ma Y.T., Sluka K.A. (2001). Reduction in inflammation-induced sensitization of dorsal horn neurons by transcutaneous electrical nerve stimulation in anesthetized rats. Exp. Brain Res..

[34] Baptista A.F., Gomes J.R., Oliveira J.T., Santos S.M., Vannier-Santos M.A., Martinez A.M. (2008). High- and low-frequency transcutaneous electrical nerve stimulation delay sciatic nerve regeneration after crush lesion in the mouse. J. Peripher. Nerv. Syst..

[35] Bersch I., Fridén J. (2021). Electrical stimulation alters muscle morphological properties in denervated upper limb muscles. Ebiomedicine.

[36] Bahadori S., Immins T., Wainwright T.W. (2017). The effect of calf neuromuscular electrical stimulation and intermittent pneumatic compression on thigh microcirculation. Microvasc. Res..

[37] Al-Zamil M., Minenko I.A., Kulikova N.G., Alade M., Petrova M.M., Pronina E.A., Romanova I.V., Narodova E.A., Nasyrova R.F., Shnayder N.A. (2022). Clinical experience of high frequency and low frequency TENS in treatment of diabetic neuropathic pain in Russia. Healthcare.

[38] Al-Zamil M.K., Kulikova N.G. (2021). Transcutaneous electroneurostimulation in treatment patients with diabetic neuropathy. Russ. J. Physiother. Balneol. Rehabil..

[39] Al-Zamil M.K., Minenko I.A. (2016). Algorithm for the treatment of carpal syndrome in patients with type 2 diabetes mellitus using acupuncture and transcutaneous electrical nerve stimulation in combination with drug therapy. Bull. New Med. Technol. Electron. Ed..

[40] Forst T., Nguyen M., Forst S., Disselhoff B., Pohlmann T., Pfützner A. (2004). Impact of low frequency transcutaneous electrical nerve stimulation on symptomatic diabetic neuropathy using the new Salutaris device. Diabetes Nutr. Metab..

[41] Sears E.D., Chung K.C. (2010). Validity and responsiveness of the Jebsen-Taylor Hand Function Test. J. Hand Surg. Am..

[42] Lin T.Y., Chang K.V., Wu W.T., Özçakar L. (2022). Ultrasonography for the diagnosis of carpal tunnel syndrome: An umbrella review. J. Neurol..

[43] Tu I.T., Jou I.M., Ko P.Y., Lee J.S., Kuo L.C., Li C.Y., Wu P.T. (2022). Diagnosis of carpal tunnel syndrome in patients without diabetes with hemodialysis using ultrasonography: Is it a useful adjunctive tool?. Arch. Phys. Med. Rehabil..

[44] Ng A.W.H., Griffith J.F., Tsoi C., Fong R.C.W., Mak M.C.K., Tse W.L., Ho P.C. (2021). Ultrasonography Findings of the Carpal Tunnel after Endoscopic Carpal Tunnel Release for Carpal Tunnel Syndrome. Korean J. Radiol..

[45] Pace V., Marzano F., Placella G. (2023). Update on surgical procedures for carpal tunnel syndrome: What is the current evidence and practice? What are the future research directions?. World J. Orthop..

[46] Jebsen R.H., Taylor N., Trieschmann R.B., Trotter M.J., Howard L.A. (1969). An objective and standardized test of hand function. Arch. Phys. Med. Rehabil..

[47] Somers D.L., Clemente F.R. (2009). Contralateral high or a combination of high- and low-frequency transcutaneous electrical nerve stimulation reduces mechanical allodynia and alters dorsal horn neurotransmitter content in neuropathic rats. J. Pain.

[48] Vance C.G., Radhakrishnan R., Skyba D.A., Sluka K.A. (2007). Transcutaneous electrical nerve stimulation at both high and low frequencies reduces primary hyperalgesia in rats with joint inflammation in a time-dependent manner. Phys. Ther..

[49] Yuan C.S., Attele A.S., Dey L., Lynch J.P., Guan X. (2002). Transcutaneous electrical acupoint stimulation potentiates analgesic effect of morphine. J. Clin. Pharmacol..

[50] Astokorki A.H.Y., Mauger A.R. (2017). Transcutaneous electrical nerve stimulation reduces exercise-induced perceived pain and improves endurance exercise performance. Eur. J. Appl. Physiol..

[51] Han J.S., Chen X.H., Sun S.L., Xu X.J., Yuan Y., Yan S.C., Hao J.X., Terenius L. (1991). Effect of low- and high-frequency TENS on Met-enkephalin-Arg-Phe and dynorphin A immunoreactivity in human lumbar CSF. Pain.

[52] Francis R.P., Johnson M.I. (2011). The characteristics of acupuncture-like transcutaneous electrical nerve stimulation (acupuncture-like TENS): A literature review. Acupunct. Electrother. Res..

[53] Wang X., Chan S.T., Fang J., Nixon E.E., Liu J., Kwong K.K., Rosen B.R., Hui K.K. (2013). Neural encoding of acupuncture needling sensations: Evidence from a FMRI study. Evid. Based Complement. Alternat Med..

[54] Han J.S. (2003). Acupuncture: Neuropeptide release produced by electrical stimulation of different frequencies. Trends Neurosci..

[55] Al-Zamil M.K., Kulikova N.G. (2021). TENS and acupuncture in treatment of carpal tunnel syndrome. Int. J. Pharmacogn. Chin. Med..

[56] Gozani S.N. (2019). Remote analgesic effects of conventional transcutaneous electrical nerve stimulation: A scientific and clinical review with a focus on chronic pain. J. Pain Res..

[57] Dailey D.L., Rakel B.A., Vance C.G. (2013). Transcutaneous electrical nerve stimulation reduces pain, fatigue and hyperalgesia while restoring central inhibition in primary fibromyalgia. Pain.

[58] Kulikova N.G., Konchugova T.V., Astakhova K.A., Nesterova E.V., Al-Zamil M.K. (2021). Indicators of bioelectrical activity of the brain in patients with distal polyneuropathy after the use of transcutaneous methods of electrical nerve stimulation of the median nerves. Quest. Curortol. Physiother. Ther. Phys. Cult..

[59] Sherry J.E., Oehrlein K.M., Hegge K.S., Morgan B.J. (2001). Effect of burst-mode transcutaneous electrical nerve stimulation on peripheral vascular resistance. Phys. Ther..

[60] Sandberg M.L., Sandberg M.K., Dahl J. (2007). Blood flow changes in the trapezius muscle and overlying skin following transcutaneous electrical nerve stimulation. Phys. Ther..

[61] do Carmo Almeida T.C., Dos Santos Figueiredo F.W., Barbosa Filho V.C., de Abreu L.C., Fonseca F.L.A., Adami F. (2018). Effects of transcutaneous electrical nerve stimulation on proinflammatory cytokines: Systematic review and meta-analysis. Mediat. Inflamm..

[62] Beckwée D., De Hertogh W., Lievens P., Bautmans I., Vaes P. (2012). Effect of tens on pain in relation to central sensitization in patients with osteoarthritis of the knee: Study protocol of a randomized controlled trial. Trials.

[63] Haastert-Talini K., Grothe C. (2013). Electrical stimulation for promoting peripheral nerve regeneration. Int. Rev. Neurobiol..

[64] Mendonça A.C., Barbieri C.H., Mazzer N. (2003). Directly applied low intensity direct electric current enhances peripheral nerve regeneration in rats. J. Neurosci. Methods.

[65] Gordon T. (2016). Electrical stimulation to enhance axon regeneration after peripheral nerve injuries in animal models and humans. Neurotherapeutics.

[66] Almeida C.C., Silva V.Z.M.D., Júnior G.C., Liebano R.E., Durigan J.L.Q. (2018). Transcutaneous electrical nerve stimulation and interferential current demonstrate similar effects in relieving acute and chronic pain: A systematic review with meta-analysis. Braz. J. Phys. Ther..

[67] Gibson W., Wand B.M., O’Connell N.E. (2017). Transcutaneous electrical nerve stimulation (TENS) for neuropathic pain in adults. Cochrane Database Syst. Rev..

[68] Al-Zamil M.K., Kulikova N.G., Vasileva E.S., Ephimov M.A. (2021). Dynamics of allodynia in the treatment of patients with diabetic polyneuropathy using transdermal electrical nerve stimulation. Russ. J. Physiother. Balneol. Rehabil..

[69] Peng W.W., Tang Z.Y., Zhang F.R., Li H., Kong Y.Z., Iannetti G.D., Hu L. (2019). Neurobiological mechanisms of TENS-induced analgesia. Neuroimage.

[70] Alarcón J.B., Chuhuaicura P.B., Sluka K.A., Vance C.G.T., Fazan V.P.S., Godoy K.A., Fuentes R.E., Dias F.J. (2022). Transcutaneous Electrical Nerve Stimulation in Nerve Regeneration: A Systematic Review of In Vivo Animal Model Studies. Neuromodulation.

[71] Su H.L., Chiang C.Y., Lu Z.H., Cheng F.C., Chen C.J., Sheu M.L., Sheehan J., Pan H.C. (2018). Late administration of high-frequency electrical stimulation increases nerve regeneration without aggravating neuropathic pain in a nerve crush injury. BMC Neurosci..

[72] Ni L., Yao Z., Zhao Y., Zhang T., Wang J., Li S., Chen Z. (2023). Electrical stimulation therapy for peripheral nerve injury. Front. Neurol..

